# Diversity and effects of competitive *Trichoderma* species in *Ganoderma lucidum*–cultivated soils

**DOI:** 10.3389/fmicb.2022.1067822

**Published:** 2022-12-08

**Authors:** Yongjun Wang, Linzhou Zeng, Jiayi Wu, Hong Jiang, Li Mei

**Affiliations:** ^1^Department of Forest Protection, College of Forestry and Biotechnology, Zhejiang A&F University, Hangzhou, China; ^2^Department of Silviculture, College of Forestry and Biotechnology, Zhejiang A&F University, Hangzhou, China

**Keywords:** species diversity, adverse effect, cultivation obstacle, volatile compounds, *Ganoderma lucidum*, *Trichoderma* spp.

## Abstract

*Ganoderma lucidum* (GL) is a well-known medicinal mushroom that has been extensively cultivated. Our previous study has shown that abundant *Trichoderma* colonies grow on the casing soil surface, posing cultivation obstacles for GL. However, an understanding of species-level characteristics of *Trichoderma* strains and their adverse effects on GL growth is limited. This study aimed to investigate the diversity and potential effects of *Trichoderma* from GL-cultivated soils. Over 700 *Trichoderma* isolates were collected from two trails in Longquan Country, southeast China. Eight *Trichoderma* species, including *T. atrioviride*, *T. guizhouense*, *T. hamatum*, *T. harzianum*, *T. koningiopsis*, *T. pleuroticola*, *T.* sp. *irale*, and *T. virens*, were identified based on the combination alignment of *tef-1α* and *rpb2* sequences. The number of *Trichoderma* colonies increased dramatically during GL cultivation, with an increase of 9.2-fold in the Lanju trail. *T. virens* accounted for the most colonies (33.33 and 32.50% in Lanju and Chengbei, respectively) at the end of GL cultivation. The *Trichoderma* species growth varied but was satisfactory under different temperature or pH conditions. Moreover, *Trichoderma* species showed different adverse effects on GL growth. The non-volatile metabolites from *T. virens* and volatile metabolites from *T. atroviride* displayed the strongest antagonistic activity. Furthermore, the volatile 6-pentyl-2H-pyran-2-one (6-PP) showed a significant inhibitory effect on GL growth with an 8.79 μl mL^−1^ headspace of 50% effective concentration. The different *Trichoderma* spp. produced different amounts of 6-PP. The most efficient 6-PP producer was *T. atroviride*. To the best of our knowledge, this study is the first to demonstrate the abundance of competitive *Trichoderma* species associated with GL cultivation. Our results would contribute to.

## Introduction

*Ganoderma lucidum* (GL, commonly known as ‘Lingzhi’ in Chinese or ‘Reishi’ in Japanese) is a traditional medicinal mushroom, which has been consumed for a long time for its high nutritive and medicinal properties (Cao et al., 2012). GL and its byproducts have multiple biological functions, including melanin synthesis inhibition and gut microbiota regulation, and possess anti-inflammatory and anti-tumor properties (Bishop et al., 2015; Chang et al., 2015). These diverse nutraceutical and pharmacological effects are mainly attributed to the high triterpenoid and polysaccharide contents in GL spores and fruiting bodies (Boh et al., 2007). Owing to its limited production in nature, GL has been artificially cultivated extensively, especially in China. Among the GL cultivation regions, Longquan County in Zhejiang Province is a famous geo-herbalism region owing to its long history of cultivation (Li et al., 2016).

GL is a wood-degrading basidiomycete that utilizes lignin as its preferred carbon source in nature (Liu et al., 2012). Short wood logs have been cultivated in Longquan County and other regions of China for a high yield of spores containing abundant active triterpenoids and polysaccharides (Zhang et al., 2004). The GL-inoculated wood logs are transferred to a mushroom house and embedded in the soil. Two harvests of GL spores within 2 years can be achieved with one cultivation season (Zhou, 2017). However, GL cultivation is hindered due to the occurrence of large amounts of competitive fungi, such as *Trichoderma*, during cultivation, incurring loss to local farmers (Tong et al., 2020). Some *Trichoderma* species, such as *T. harzianum* and *T. atroviride*, cause green mold disease in cultivated GL (Lu et al., 2016; Yan et al., 2019).

The genus *Trichoderma* Pers. (Ascomycetes, Hypocreales) is cosmopolitan and found in soil or decaying woods (Schuster and Schmoll, 2010). Many species in this genus are economically and ecologically important. The species *T. harzianum* and *T. virens* are biostimulants or biocontrol agents applied in the field (Harman, 2006). *T. reesi* is a well-known industrial cellulose producer (Bischof et al., 2016). Some species, such as *T. longibrachiatum* and *T. virens*, are used to remediate soil and water pollution (Babu et al., 2014; Andreolli et al., 2016). However, a few species are causal agents of green mold disease in mushroom cultivation (Komon-Zelazowska et al., 2007; Kim et al., 2012; An et al., 2022). Hatvani et al. (2007) reported that *T. aggressivum* is an epidemic species causing green mold disease on champignon (*Agaricus bisporus*) and oyster mushrooms (*Pleurotus ostreatus*) in Europe. Six *Trichoderma* species, including *T. harzianum*, *T. atroviride*, *T. viride*, *T. pleuroticola*, *T. longibrachiatum*, and *T. oblongisporum*, have been associated with green mold disease in Shiitake (*Lentinula edodes*) in China (Wang et al., 2016). The species *T. pleuroticola* and *T. pleuroti* have been associated with green mold diseases in champignon mushrooms in America (Royse et al., 1999; Innocenti et al., 2019).

Our previous investigation revealed that the accumulation of antagonistic fungi, mainly *Trichoderma*, contributed to GL cultivation problems in Longquan County, China (Tong et al., 2020). This finding provided a basis for determining *Trichoderma* biodiversity in Longquan County and its competitive effects on GL growth. However, the species-level population dynamic of *Trichoderma* strains during GL cultivation and their potential effects on GL growth are still unknown. Therefore, this study aimed to investigate the diversity and effect of *Trichoderma* strains on GL-cultivated soils. We collected *Trichoderma* isolates and identified their species based on sequence analyzes of the combined partial sequences of translation elongation factor 1-alpha (*tef1*-α) and the second largest RNA polymerase subunit encoding genes (*rpb2*). The population changes of *Trichoderma* colonies on the surface of GL-cultivated soils during cultivation were also investigated. The results provide information on *Trichoderma* diversity and its antagonistic characteristics on GL development.

## Materials and methods

### *Ganoderma lucidum* cultivation

**Figure 1:**
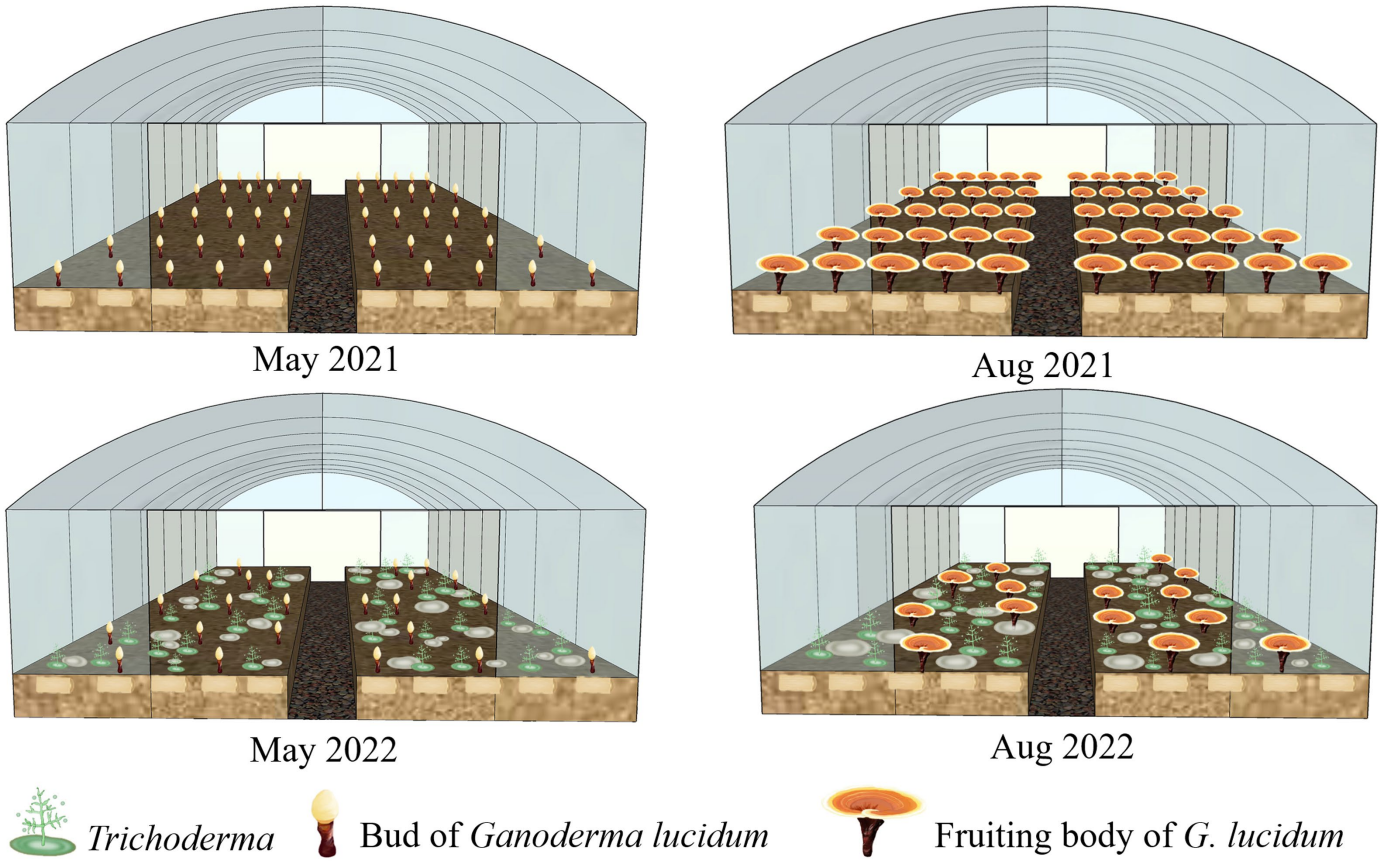
An illustrative scheme of *Ganoderma lucidum* cultivation in Longquan County of China and the sampling period.

GL Hu-nongke 1, widely cultivated in Longquan County, Zhejiang Province, China, was used in this study for GL production by the short wood-log cultivation method as described previously (Tong et al., 2020). The wood logs from a local sawtooth oak (*Quercus acutissima*) were used for GL cultivation. The GL-inoculated wood logs were embedded in the soil of mushroom houses (10 m width × 50 m length), as shown in Figure 1.

### Collection and isolation of *Trichoderma* colonies

The fungal samples were scraped and labeled from *Trichoderma* colonies on the surface of GL-cultivated soils in two trails, Lanju (N27^°^57′55″, E119^°^02′31″) and Chengbei (N28^°^13′22″, E119 ^°^04′34″), in Longquan County, southeast China. Field sampling was conducted in May and August of 2021 and 2022 (Figure 1). *Trichoderma* strains were isolated and maintained as described previously (Hatvani et al., 2007). All isolates were deposited in the culture collection of the Department of Forest Protection, Zhejiang A&F University, China. The *Trichoderma* isolates were inoculated on 9 cm-diameter Petri dishes containing potato dextrose agar (PDA; 200 g potato, 20 g glucose, 18 g agar, 1 l H_2_O) or cornmeal dextrose agar (CMD; Oxoid, Hampshire, United Kingdom) at 26°C. Morphological characteristics of the colonies were recorded. Microscopic characteristics were observed under a Motic M200 microscope (Motic, China).

### DNA extraction, PCR amplification, and DNA sequencing

Fungal DNA was extracted using the Ezup Column Fungi Genomic DNA Purification Kit (Sangon Biotech, Shanghai, China), following the manufacturer’s instructions. The primers used to amplify the *tef-1α* and *rpb2* genes from *Trichoderma* were: tef1-F (5′-CATCGAGAAGTTCGAGAAGG-3′), tef1-R (5′-AACTTGCAGGCAATGTGG-3′), rpb2-F (5′-TGGGGWGAYCARAARAAGG-3′), and rpb2-R (5′-CATRATGACSGAATCTTCCTGGT-3′) (Cai and Druzhinina, 2021). The PCR amplification products were separated using electrophoresis in 1.0% (w/v) agarose gels. The purified PCR products were sent to Tsingke Biotechnology Co., Ltd. for Sanger sequencing. The obtained sequences were deposited in the GenBank database at the National Center for Biotechnology Information (NCBI), and their accession numbers are listed in Supplementary Table S1.

### Phylogenetic analysis and identification

Nucleotide quality and contig assembly were performed using Staden Package v.2.0.0b11 (Staden et al., 2000). The DNA sequence dataset was constructed using MEGA v.7.0 (Kumar et al., 2016). The aligned *tef1-1α* and *rpb2* gene sequences of all *Trichoderma* isolates were used for BLAST interface analysis and alignment in the NCBI database.^1^ After identification, the aligned sequences were submitted to the NCBI GenBank to obtain accession numbers. Molecular phylogenetic analysis between these selected fungi with our strains was performed using the maximum-likelihood method. Nodal robustness was tested using the bootstrap method, and phylogenetic robustness was determined using MEGA v.7.0 with 1,000 replications (Kumar et al., 2016). *Protocrea pallida* CBS299.78 was used as an outgroup to root the tree.

### *Trichoderma* population assay

*Trichoderma* population assays were conducted in the two trails: Lanju and Chengbei. *Trichoderma* colonies grown on the surface of GL-cultivated soil from three randomly chosen mushroom houses in each trail were collected and counted from 2021 to 2022. The numbers of *Trichoderma* colonies were individually recorded at the time points in April and August each year. The species affiliation of each *Trichoderma* colony was determined by molecular identification, as described above.

### Effects of temperature on mycelial growth of *Trichoderma* strains

One representative strain for each of the eight *Trichoderma* species was selected to test the effect of different temperatures on fungal mycelial growth: *T. atroviride* LZ043, *T. hamatum* LZ007, *T. harzianum* LZ013, *T. koningiopsis* LZ033, *T. pleuroticola* LZ004, *T. guizhouense* LZ056, *T. spirale* LZ023, and *T. virens* LZ045. A 5 mm-diameter mycelial plug was inoculated in a PDA plate and cultured separately at 13, 23, 31, and 35°C. Colony diameter was measured every day using a Vernier caliper. Each treatment was replicated three times. The assays were conducted using two biological replicates.

### Effects of pH on mycelial growth of *Trichoderma* strains

The eight representative strains were used to test the effect of different pH levels on mycelial growth. Before inoculation, the pH values of PDA were individually adjusted to 4.5, 5.5, 6.5, and 7.5 by adding HCl or NaOH solution filtered through a biofilter after sterilization. A 5 mm-diameter mycelial plug was inoculated into the PDA plate and cultured at 26°C. Colony diameter was measured using a Vernier caliper. Each treatment was replicated three times. The assays were conducted using two biological replicates.

### Effects of *Trichoderma* strains on GL mycelia in petri plates

The eight representative strains were chosen to test the antagonistic potential of the *Trichoderma* species using the dual confrontation method. Mycelial agar plugs (5 mm in diameter) of GL and each *Trichoderma* strain were cut individually from the actively growing front of 7-day-old colonies. Each *Trichoderma* plug was paired against GL at equal distances opposite to each in 90 mm diameter Petri plates containing 20 ml PDA. The plates were incubated at 26°C in darkness for 7 days. The GL growth were recorded. The experiments were carried out with three replicates. The assays were conducted using two biological replicates.

### Effects of non-volatile metabolites from different *Trichoderma* strains on GL hyphal growth

The fermentation broths from eight representative *Trichoderma* strains were used to test the effects of non-volatile metabolites from different *Trichoderma* spp. on GL growth. The *Trichoderma* fermentation broth was prepared as previously described (Wang et al., 2016). Briefly, a 5 mm-diameter plug from each *Trichoderma* culture on PDA was inoculated in 500 ml PD broth, followed by 7 days culturing in darkness at 26°C at 180 rpm. Each *Trichoderma* culture was filtered sequentially through cheesecloth, Whatman filter paper, and a 0.2 μm nitrocellulose filter. The filtrates were added to the melted PDA to a volume of 10%; the same volume of sterile water was used as a control. A GL mycelium plug (5 mm in diameter) was inoculated into the medium and cultured in darkness at 26°C. After 7 d, the diameters of the colonies were measured to test the inhibition ratios. The percent inhibition ratio (*IR*) was computed by the formula: IR = [(*R1*-*R2*) /*R1*] × 100, Where *R1* is radial growth of GL in control; *R2* is radial growth of GL in treatment. Each treatment was replicated three times. The assays were conducted using two biological replicates.

### Effects of volatiles emitted by different *Trichoderma* strains on GL hyphal growth

The sandwich Petri plate setup described previously was employed to test the antagonistic activity of the volatiles emitted by the representative *Trichoderma* strains against GL (Li et al., 2018). After inoculating GL and *Trichoderma* on PDA plates, the GL plate was placed on top of a *Trichoderma* plate, sealed with three layers of Parafilm, and then incubated at 26°C. A non-inoculated PDA plate was used as the control. GL colony diameters were measured after 5 days. The percent inhibition ratio (*IR*) was computed by the formula: *IR* = [(*R1*-*R2*) /*R1*] × 100, Where *R1* is radial growth of GL in control; *R2* is radial growth of GL in treatment. Each treatment included two biological replicates and was repeated three times.

### Analysis of the volatile contents produced by different *Trichoderma* strains

Solid-phase microextraction coupled with gas chromatography–tandem mass spectrometry (SPME–GC–MS) was used to determine the composition of the volatiles produced by the representative *Trichoderma* strains (Stoppacher et al., 2010). The detailed procedure and conditions have been described previously (Rao et al., 2022). *Trichoderma* was cultured on PDA and incubated at 26°C for 5 days. SPME inserted into the injection port of a GC 2010 gas chromatograph was used to collect the volatiles. SPME fiber was exposed to the vapor phase above strain LZ42 for 45 min in a culture tube. A Hewlett Packard 7890GC/5975MSD gas chromatograph (Agilent Technologies, Santa Clara, CA, United States) equipped with an HP-5MS capillary column was used. The derived data were analyzed and identified based on a comparison with the mass spectrum in the NIST08.L data bank (National Institute of Standards and Technology). Each experiment was conducted in triplicates. Pure volatile 6-pentyl-2H-pyran-2-one (6-PP, Sigma-Aldrich, St. Louis, MO, United States) was used as the standard.

### Antifungal activity assay of the volatile 6-PP

The antifungal activity of volatile 6-PP on GL was tested by fumigation in an isolated container, as described previously (Wu et al., 2020). The EC_50_ value of 6-PP on GL was calculated as the 50% effective concentration that inhibits GL mycelial growth using the IBM SPSS Statistics v.19 program (SPSS, Inc., Chicago, IL, United States). A 5 mm-diameter mycelial plug was placed in the center of the PDA. Meanwhile, the 6-PP aliquots of 1, 2, 4, 8, 16, and 32 μl were individually loaded on the filter paper (60 mm in diameter) before incubation, resulting in 0.01, 0.03, 0.05, 0.11, 0.21, and 0.43 μl mL^−1^ headspace, respectively. Subsequently, the loaded plates were immediately sealed with Parafilm and incubated at 26°C for 5 days. The assays were conducted in triplicate, and plates with filter paper alone were used as controls.

### Statistical analysis

The obtained data were analyzed using the IBM SPSS Statistics 20.0 (SPSS Inc.). Statistical significance was identified at 95% confidence interval (*p* < 0.05) in all tests.

## Results

### Sample collection and identification of *Trichoderma* spp. in GL mushrooms

**Figure 2:**
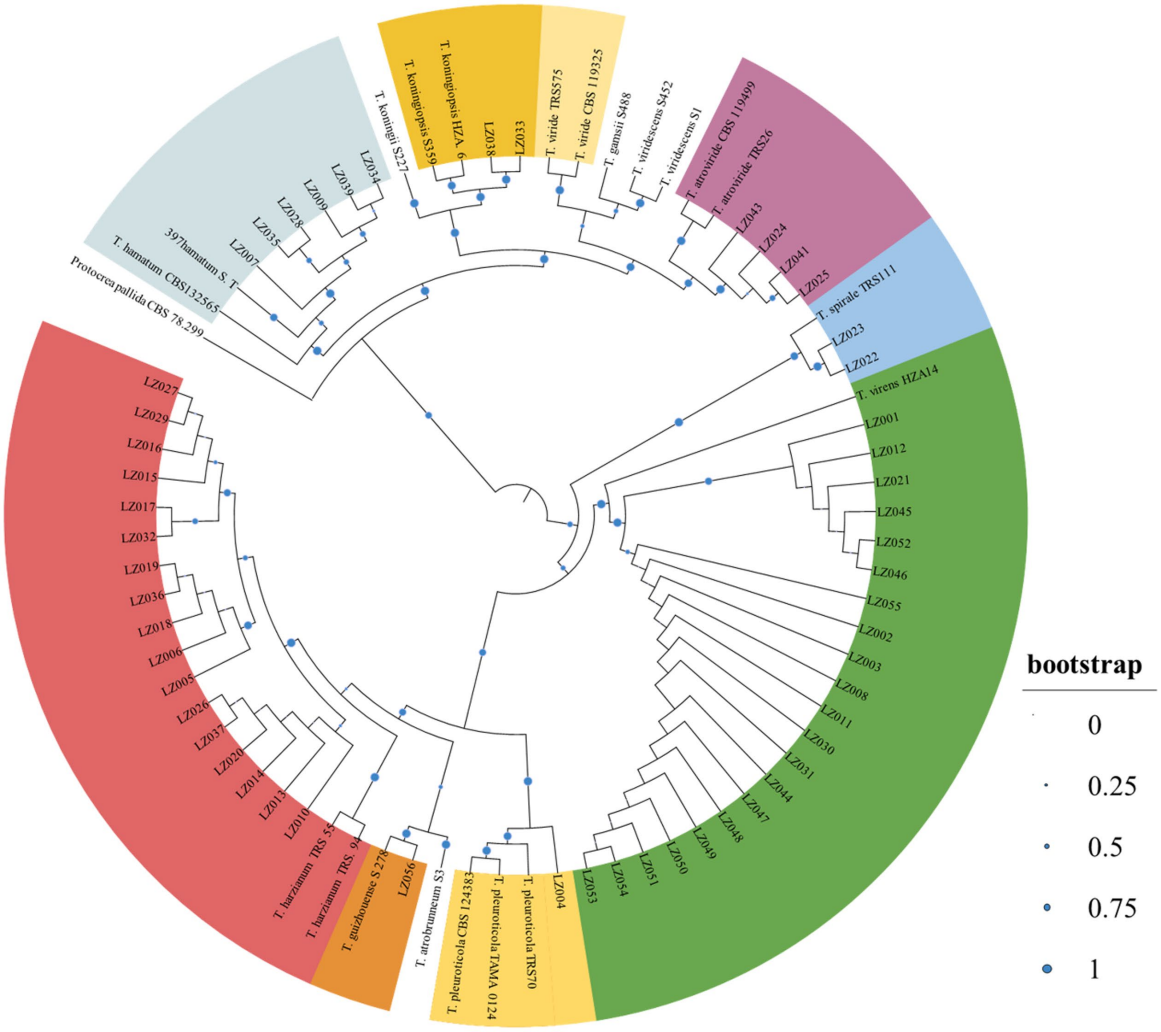
Phylogenetic tree of the representative *Trichoderma* isolates using the maximum-likelihood method based on the condensed *tef-1α* and *rpb2*. The tree was constructed using Mega 7.0 with 1,000 bootstrap replications condensed with a 70% cut-off value and viewed on iTOL. *Protocrea pallid*a CBS 78.299 was used as an outgroup.

During the investigation period, *Trichoderma* colonies began appearing on the soil surface of cultivation ridges in the mushroom houses in April 2021 when the sprouts of GL fruiting bodies developed (Figure 1). From the GL mushroom houses, 701 and 685 *Trichoderma* isolates were collected after 2-year GL cultivation in the Lanju and Chengbei trails, respectively. These isolates presented diverse morphological characteristics (data not shown). A phylogenetic tree of the 73 representative *Trichoderma* isolates with different morphological characters were constructed using the maximum-likelihood method based on the combination of *tef-1α* and *rpb2* sequences (Figure 2). The combined sequence matrix contained 2,271 bp (1,118 for *tef1-α* and 1,031 for *rpb2*). Eight *Trichoderma* species were identified among these isolates with those that were previously documented as threshold of species: *T. atrioviride*, *T. guizhouense*, *T. hamatum*, *T. harzianum*, *T. koningiopsis*, *T. pleuroticola*, *T. spirale*, and *T. virens*.

### Population changes of *Trichoderma* spp. during GL cultivation

**Figure 3:**
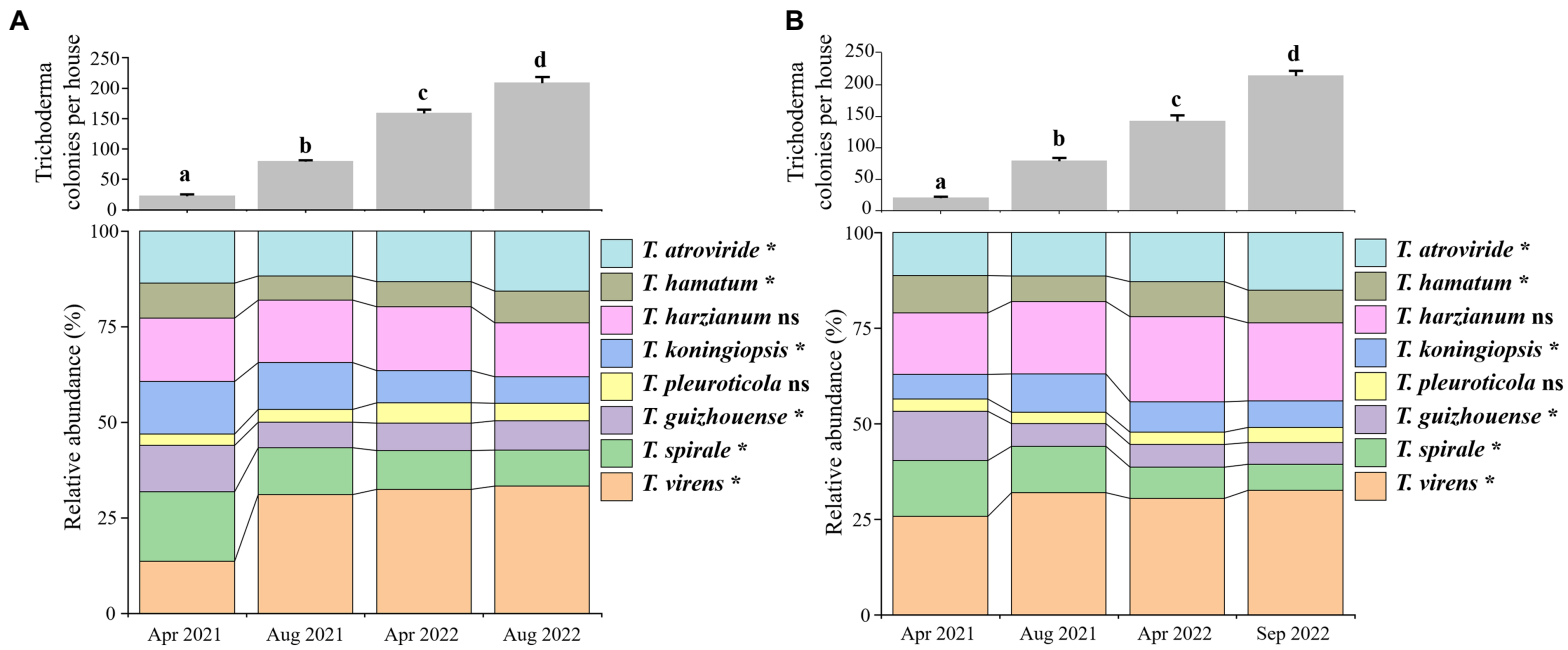
Population changes of *Trichoderma* spp. on the soil surface of *Ganoderma lucidum* cultivation houses in Lanju **(A)** and Chengbei **(B)** trails. The bar graphs show the increase in the colony number of all *Trichoderma* species on the soil surface per house. Data were the average number of *Trichoderma* colonies in six mushroom houses. Means in the plot topped by the different letters indicate the significant difference based on Duncan’s multiple range test of one-way ANOVA at *p* < 0.05. The lower panels show the taxonomic composition of *Trichoderma* communities in the different investigation periods at the species level. ns, not significant at *p* < 0.05; asterisk indicates significance at *p* < 0.05 based on Games–Howell post-hoc test and Benjamini–Hochberg false discovery rate (FDR) as the multiple test correction methods.

*Trichoderma* propagation on the surfaces of GL-cultivated soils was analyzed individually in the Lanju and Chengbei trails since April 2021, based on colony sampling and identification of *Trichoderma* isolates. The colonies of *Trichoderma* spp. in both trails started appearing in April 2021 and increased significantly thereafter (Figure 3). In the Lanju trail, the average number of *Trichoderma* colonies in each house was 21 in April 2021 but increased by 2.8-fold in August 2021 and 5.8-fold in April 2022. The average number of *Trichoderma* colonies reached 214 in August 2022, an increase of 9.2-fold. Similarly, dynamic changes in *Trichoderma* colonies were observed in the Chengbei trail.

Moreover, population and proportion changes of the eight *Trichoderma* species in both trails were obtained (Figures 3A,B). Significant differences were observed in *Trichoderma* community during GL cultivation. *T. virens* was the most dominant species at the end of GL cultivation, accounting for 32.50 and 33.33% of the community in the Lanju and Chengbei trails, respectively. *T. harzianum* accounted for 20.37 and 14.10% of the community in the Lanju and Chengbei trails, respectively. *T. pleuroticola* was found in the lowest amount, accounting for 3.89 and 4.65% of the community in the Lanju and Chengbei trails, respectively.

### Effects of different temperatures and pH levels on the mycelial growth of *Trichoderma* spp.

**Figure 4:**
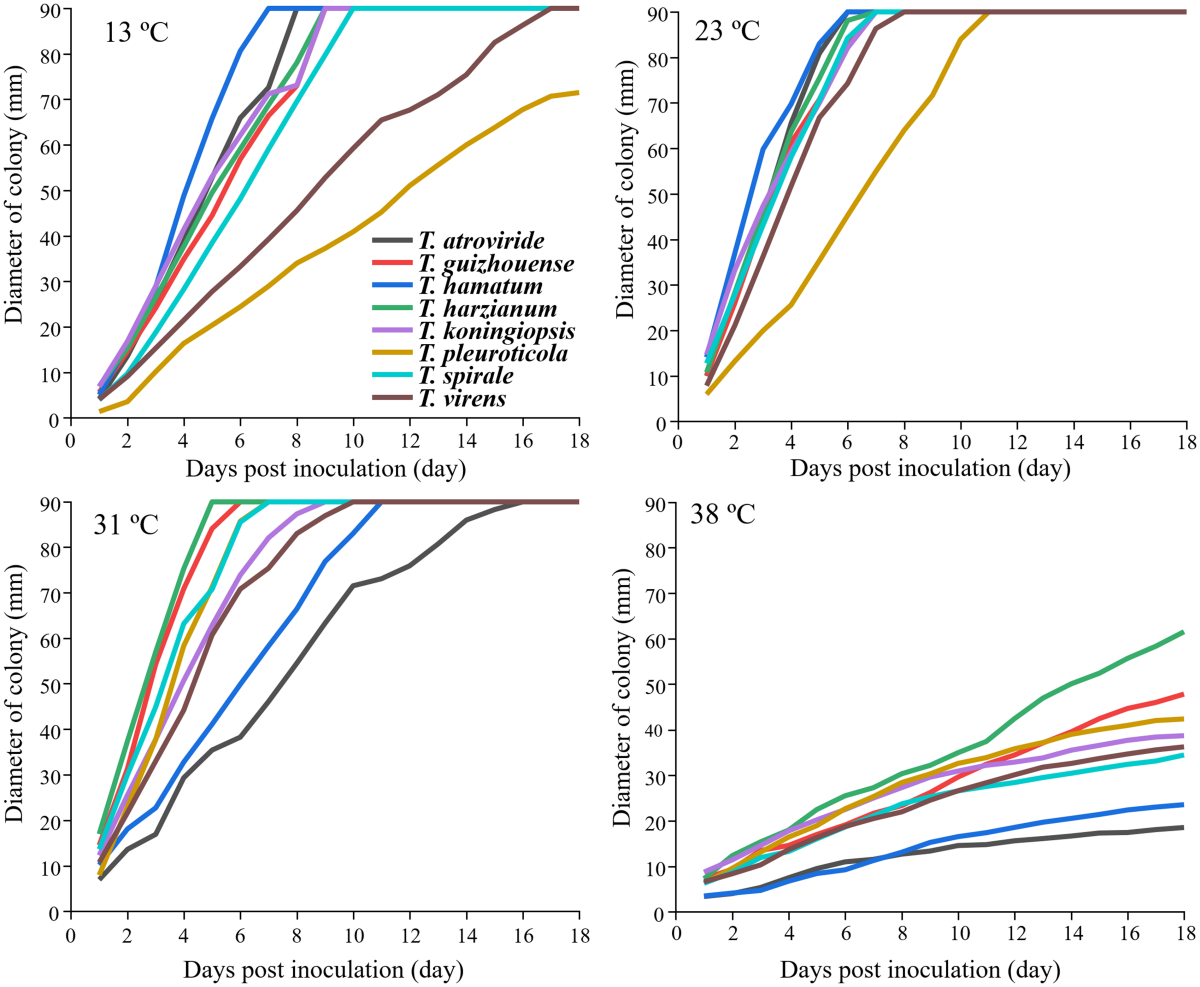
Effects of different temperatures on the mycelial growth of different *Trichoderma* strains on the PDA plates. Data represent the mean of two independent replicates with three plates each.

**Figure 5:**
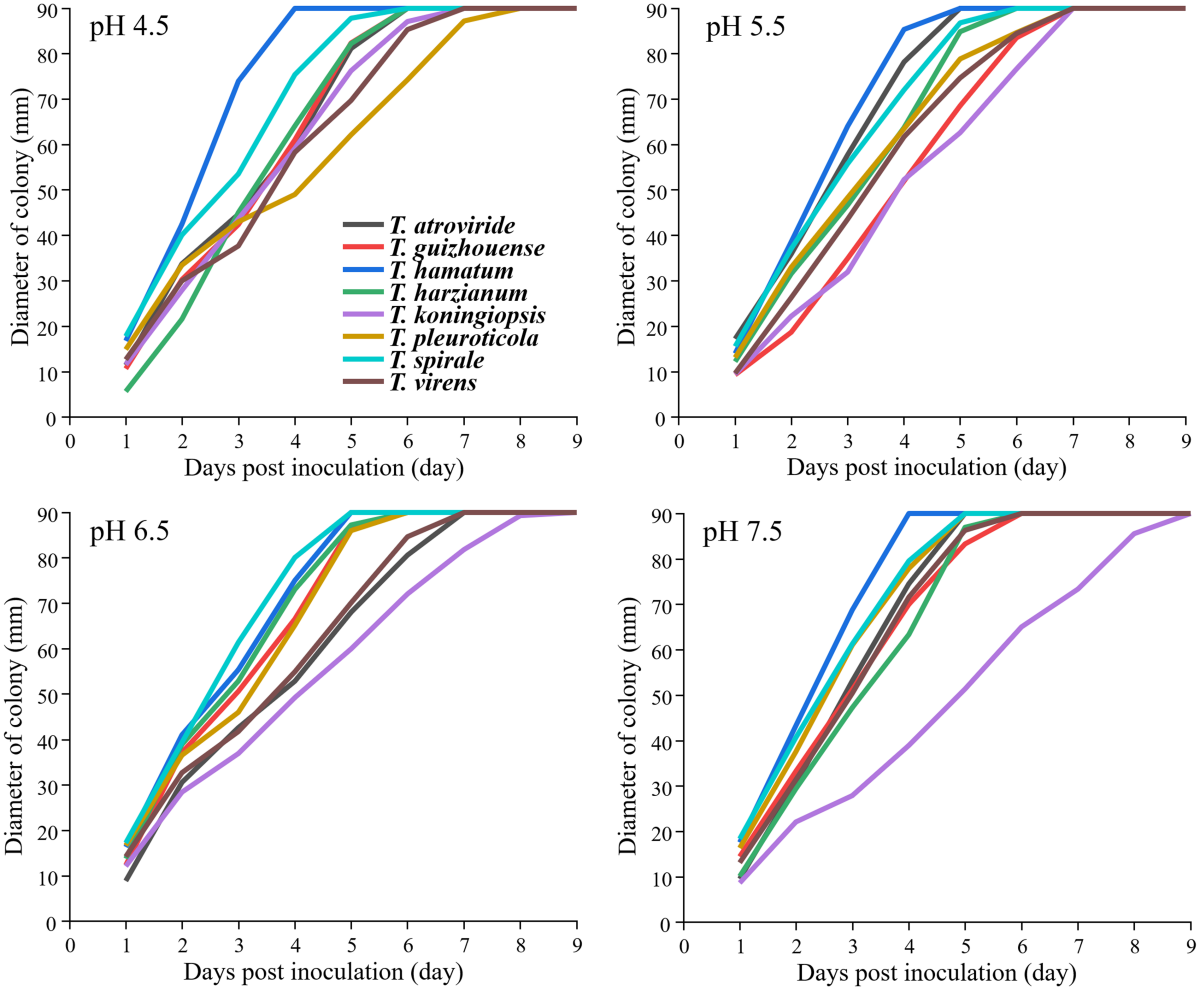
Effects of different pH levels on the mycelial growth of different *Trichoderma* strains on the PDA plates at 26°C. Data represent the mean of two independent replicates with three plates each.

The strains from the eight *Trichoderma* spp. showed different growth rates at different temperatures (Figure 4). *T. hamatum* had the highest radium length at 15°C within 7 days post-inoculation, whereas *T. pleuroticola* had the lowest radium length, followed by *T. virens*. All *Trichoderma* species grew rapidly at 23°C and 31°C. The growth rate of *T. pleuroticola* at 31°C was higher than that at 23°C. *T. harzianum* grew rapidly compared to other species at 38°C. The eight *Trichoderma* species grew normally at pH 4.5–7.5 (Figure 5). Among these species, *T. koningiopsis* exhibited the lowest growth rate at pH 4.5. Overall, the eight *Trichoderma* species isolated from GL-cultivated soils could survive over a broad range of environmental temperatures and pH values.

### Antagonistic activities of different *Trichoderma* species against GL

**Figure 6:**
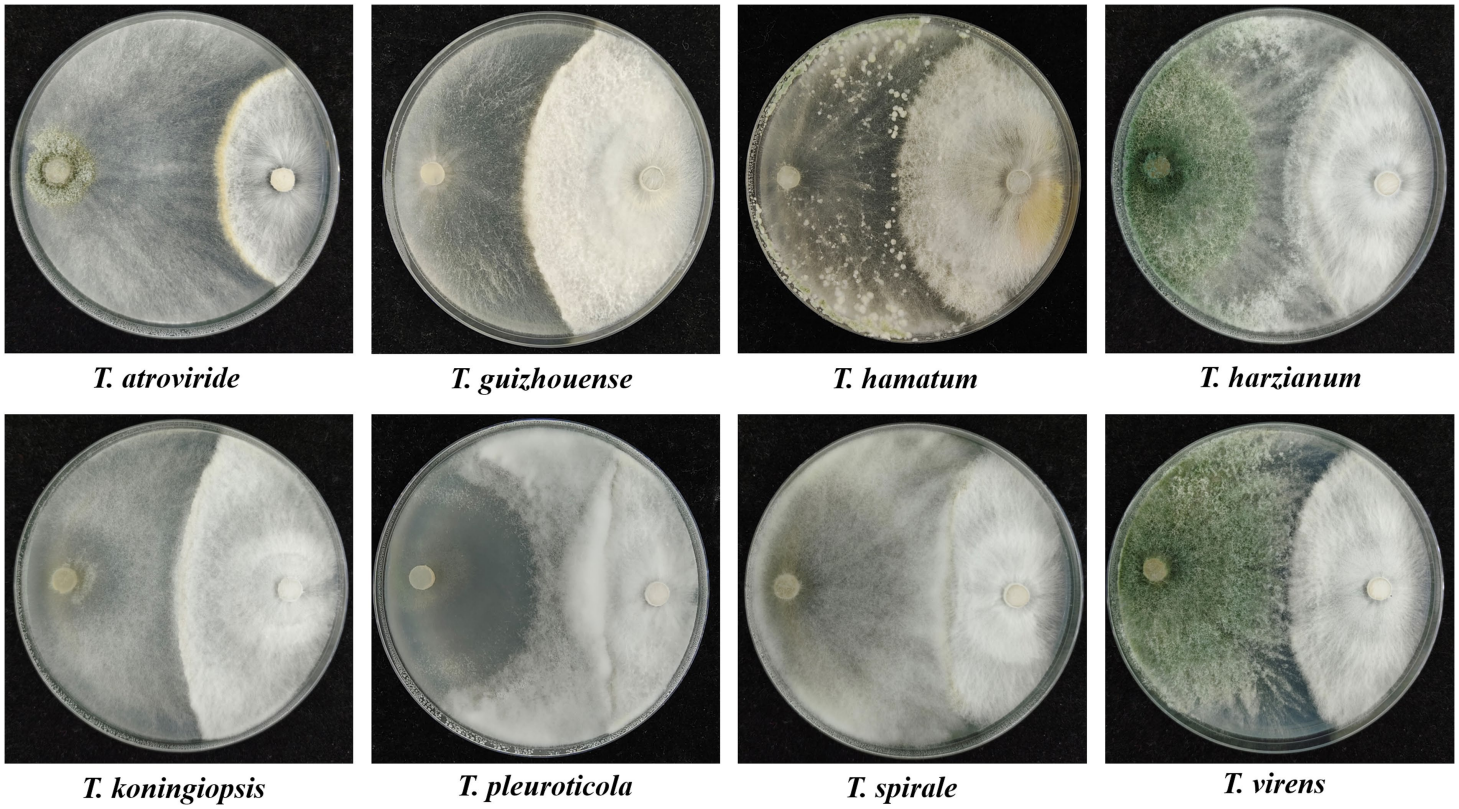
Dual cultures of the representative isolates of different *Trichoderma* species (left) against *Ganoderma lucidum* (right) on PDA plates for 6 days.

**Figure 7:**
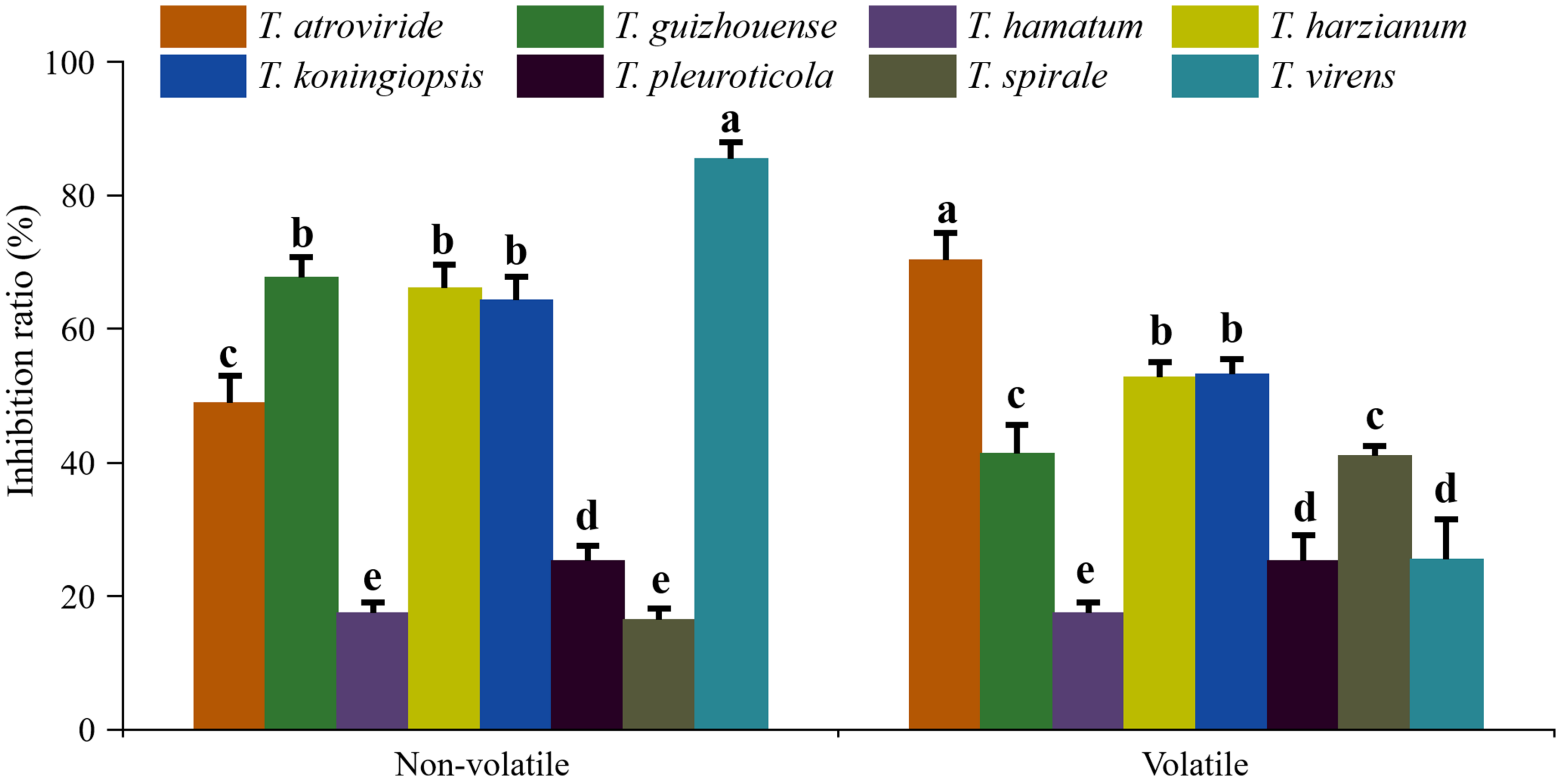
Inhibition rates of different *Trichoderma* species on the mycelial growth of *Ganoderma lucidum*. Data represent the mean of two independent replicates with five plates each. Significant differences (*p* < 0.05) between means are indicated using different letters.

In dual confrontation assays, antagonistic zones were observed in the interactions between the eight representative isolates of different *Trichoderma* species and GL on PDA plates, which indicated that all eight species exhibited antifungal activities against GL (Figure 6). *T. atroviride*, *T. harzianum* and *T. virens* inhibited heavily GL mycelium growth. The growth of GL was also inhibited by *T. guizhouense*, *T. hamatum*, or *T. koningiopsis*. *T. pleuroticola* and *T. spirale* showed less inhibitive activity, but overgrew and spread on GL mycelia. Moreover, the inhibitory effects of non-volatile and volatile metabolites on GL varied among different species (Figure 7). The non-volatile metabolites from *T. virens* showed an inhibition rate of 85.42% for GL, exhibiting the strongest antagonistic activity against GL. *T. guizhouense*, *T. harzianum*, and *T. koningiopsis* showed low activities, accounting for inhibition rates of 67.62, 66.07, and 64.29%, respectively. Meanwhile, the volatiles from *T. atroviride* inhibited the mycelial growth of GL at the highest level (70.29%), followed by *T. harzianum* and *T. koningiopsis*. The inhibition rate of *T. hamatum* was the lowest (17.4%), followed by *T. spirale*.

### Production of antifungal 6-PP in different *Trichoderma* species

**Figure 8:**
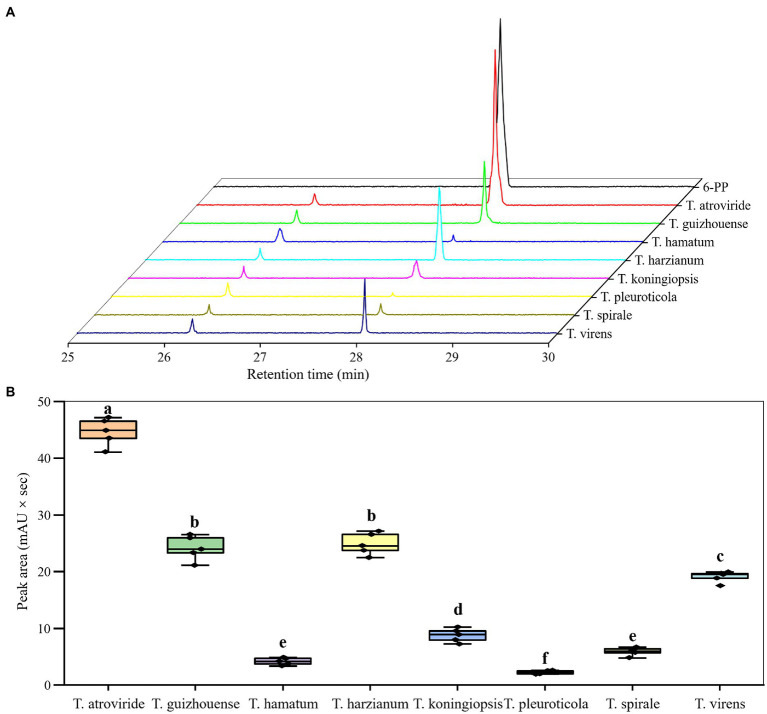
Production of 6–pentyl–2H–pyran–2–one (6-PP) by different *Trichoderma* species. **(A)** SPME–GC–MS profiles of the fungal volatiles produced by different *Trichoderma* species. **(B)** Difference peak areas of 6–PP from different *Trichoderma* species. Data represent the mean of three independent replicates. Significant differences (*p* < 0.05) between means are indicated by different letters.

In this study, the pure chemical 6-PP exhibited an inhibitory effect on GL mycelial growth (Table 1). The EC_50_ of 6-PP against GL was 8.79 μl mL^−1^. Moreover, the SPME–GC–MS analysis revealed that all the representative strains for eight *Trichoderma* species produced 6-PP, but with different levels of production (Figure 8). The differences in the peak areas showed that *T. atroviride* produced the largest amount of 6-PP, followed by *T. guizhouense* and *T. harzianum*. The production of 6-PP by *T. pleuroticola* was the lowest.

**Table 1:** Inhibitory effects of 6-pentyl-2H-pyran-2-one on mycelial growth of *Ganoderma lucidum* at 96 h post-inoculation.

Chemical	EC_50_ (μL mL^−1^ headspace)
6-pentyl-2H-pyran-2-one	8.79

## Discussion

Edible and medical mushrooms are cultivated on a large scale in many countries; it is a key production in the circle of “turn waste into treasure” of agriculture chains (Grimm and Wosten, 2018). The traditional medical mushroom GL, which has high value for human medical supply and utilization of agricultural and forest resources, has been cultivated extensively (Li and Xu, 2022). Compared to many plant crops, GL cultivation requires higher costs and more labor owing to precise management and expensive equipment (Zhou, 2017). Mushroom diseases and growth inhibitors present risks to GL production. However, competition caused by many environmental microorganisms and mushroom diseases caused by some pathogens-present serious problems in GL cultivation (Ke et al., 2019; An et al., 2022). In this study, diverse *Trichoderma* species isolated from GL-cultivated soils were investigated. Eight *Trichoderma* species, including *T. atrioviride*, *T. guizhouense*, *T. hamatum*, *T. harzianum*, *T. koningiopsis*, *T. pleuroticola*, *T. spirale*, and *T. virens*, were identified. Competitive effects of these *Trichoderma* spp. on GL growth were demonstrated. These results provided detailed information on the cultivation challenges displayed by *Trichoderma*, affecting GL production in China (Tong et al., 2020).

*Trichoderma* spp. are well-known as soil inhabitants, existing under a wide range of environmental conditions (Harman, 2006). The diversity of *Trichoderma* species in soils has been extensively studied by different researchers worldwide (Jaklitsch and Voglmayr, 2015; Jiang et al., 2016; Nawaz et al., 2018; Dou et al., 2019). Moreover, ecosystem and climatic factors significantly influence *Trichoderma* diversity (Sariah et al., 2005; Widden and Scattolin, 2018; Hu et al., 2020). During mushroom cultivation, excess exogenous carbon and nitrogen resources are released into the casing soil from the degraded culture substrate, making it a unique ecosystem (Cai et al., 2009; McGee, 2018; Carrasco et al., 2019). Plenty of *Trichoderma* strains have been isolated from soils with mushroom cultivation (Hatvani et al., 2007; Wang et al., 2016; Oh et al., 2018; Allaga et al., 2021). Seven known *Trichoderma* species and a novel species, *T. ganodermatigerum,* were identified from the soils or fruiting bodies of *G. sichuanense* in northeast China (An et al., 2022). The high diversity of *Trichoderma* and increased population changes during GL cultivation in southeast China were demonstrated in this study.

*Trichoderma* spp. exhibit antagonistic activity against a broad spectrum of microorganisms (López-Bucio et al., 2015). This highly antagonistic characteristic would contribute to their rapid colonization and propagation in ecological niches (Schuster and Schmoll, 2010). In this study, all the eight representative strains for *Trichoderma* species exhibited antagonistic activities or competitive growth toward GL. Our results further evidenced that *Trichoderma* species showed various effects against GL. Similar phenomenon had been observed on *A. bisporus* (Innocenti et al., 2019) and *L. edodes* (Wang et al., 2016). The competitive activity based on antifungal metabolites and/or enzymes production, mycoparasitism, or ecological competition, might varied for different *Trichoderma* species (Vinale et al., 2008; Anees et al., 2010; Mukherjee et al., 2012). The direct effects of these *Trichoderma* spp. were expressed as the inhibition of GL growth by non-volatile and volatile metabolites. It was well documented that *Trichoderma* produced plenty of secondary metabolites with antifungal activity, including terpenes, pyrones, gliotoxin, peptaibols, etc., (Zeilinger et al., 2016; Khan et al., 2020). This highly antagonistic function may contribute to the accumulation of their population and threaten the growth and development of GL. In contrast, the broad perspective of microbial competition and antagonism of *Trichoderma* makes it one of the front-line microorganisms commonly employed in the preventive control of different plant pathogens (Zin and Badaluddin, 2020). Our data provide a potential strategy for investigating novel *Trichoderma* bioresources for application in sustainable agriculture.

The volatile metabolites produced by different *Trichoderma* spp. were highlighted in this study. Microbial volatile metabolites, mainly volatile organic compounds (VOCs), exhibit multiple biological functions (Contreras-Cornejo et al., 2014; Kanchiswamy et al., 2015; Kottb et al., 2015; Wonglom et al., 2020). High VOC production might require screening of the isolates or modification of the culture conditions (Flores et al., 2019; Wu et al., 2020). Among the various VOCs produced by *Trichoderma* spp., 6-PP is the most common and well-known compound that exhibits a broad-spectrum antagonism (El-Hasan et al., 2016; Elsherbiny et al., 2020; Bello et al., 2022). In this study, GL growth was substantially inhibited by 6-PP. Moreover, *T. atroviride* was the most efficient producer of 6-PP on PDA among the eight species and showed the highest level of inhibition against GL. These results were consistent with the findings of Jeleń et al. (2014).

Both *T. harzianum* and *T. atroviride* are aggressive pathogens causing green mold diseases in *Ganoderma* mushrooms (Lu et al., 2016; Yan et al., 2019). Several other *Trichoderma* spp., such as *T. guizhouense*, *T. koningiopsis*, and *T. pleuroticola*, are important pathogens in other mushrooms (Innocenti et al., 2019; Chen et al., 2021). These *Trichoderma* spp. can grow under a broad range of environmental temperatures and pH levels, which is consistent with our results. Moreover, *Trichoderma* spp. utilize diverse substrates and resist noxious chemicals (Ramírez-Valdespino and Orrantia-Borunda, 2021). *Trichoderma* produces large amounts of conidia for propagation and survival (Schuster and Schmoll, 2010). These factors impede the elimination of *Trichoderma* from mushroom cultivation. Currently, the management of *Trichoderma*-associated problems depends on fungicide use, such as prochloraz manganese and thiabendazole (Pecchia and Beyer, 2013; Subedi et al., 2021). Some beneficial *Bacillus* species have been used in the disease management of *Agaricus bisporus* (Stanojevic et al., 2019). In some cases, sanitation with waterlogging reduces *Trichoderma* inocula in mushroom cultivation (Bruno et al., 2015; Tong et al., 2020). The high diversity of *Trichoderma* and their rapid population growth in GL mushrooms suggest a further evaluation to control individual species.

In conclusion, the findings of this study provide detailed information on *Trichoderma* diversity in GL-cultivated soils in China. The accumulation of these antagonistic *Trichoderma* species might be a challenge in GL cultivation. Diverse effects of different *Trichoderma* species on GL were demonstrated in this study. To the best of our knowledge, our study is the first to provide data on the abundance of *Trichoderma* species associated with continuous cultivation problems in GL production. The information obtained in this study may provide a basis for the integrated management of cultivation problems in GL. Moreover, studies on the abundant *Trichoderma* species may provide novel resources for plant-beneficial microorganisms.

## Data availability statement

The datasets presented in this study can be found in online repositories. The names of the repository/repositories and accession number(s) can be found in the article/Supplementary material.

## Author contributions

YW: conceptualization, methodology, investigation, and writing-original draft preparation. LZ: investigation, data curation, and visualization. JW: software, validation. HJ: visualization, investigation. LM: supervision and writing-reviewing and editing. All authors contributed to the article and approved the submitted version.

## Funding

This study was financially supported by a grant from the National Key Research and Development Program of China (2017YFD0201100-7) and grants from Zhejiang Key Research and Development Program of China (2019C0203002 and 2018C02034).

## Conflict of interest

The authors declare that the research was conducted in the absence of any commercial or financial relationships that could be construed as a potential conflict of interest.

## Publisher’s note

All claims expressed in this article are solely those of the authors and do not necessarily represent those of their affiliated organizations, or those of the publisher, the editors and the reviewers. Any product that may be evaluated in this article, or claim that may be made by its manufacturer, is not guaranteed or endorsed by the publisher.

## Footnotes

^1^ http://blast.ncbi.nlm.gov
