# Supplementary material for: Diversity and effects of competitive Trichoderma species in Ganoderma lucidum–cultivated soils
Source: Front Microbiol. 2022 Dec 8;13:1067822. doi: 10.3389/fmicb.2022.1067822 (PMC9772278; doi:10.3389/fmicb.2022.1067822)
Supplement: Supplementary file 1 [file Table_1.DOCX]

Supplementary Material

# Table S1. GenBank accession numbers of taxa used in phylogenetic analyses. The studied strains of *Trichoderma* were shown in bold.

| Species name | Strain number | GenBank accession number | |
| --- | --- | --- | --- |
|  |  | *tef-1α* | *rpb2* |
| *Protocrea pallida* | CBS299.78 | EU703900 | EU703948 |
| *T. atrobrunneum* | S3 | KJ665376 | KJ665241 |
| *T. atroviride* | TRS26 | KJ786832 | KP009054 |
| *T. atroviride* | CBS119499=Hypo326 | FJ860611 | FJ860518 |
| *T. atroviride* | **LZ024** | **OL957047** | **OM967099** |
| *T. atroviride* | **LZ025** | **OM967086** | **ON010779** |
| *T. atroviride* | **LZ041** | **OM967087** | **ON010780** |
| *T. atroviride* | **LZ043** | **OM967088** | **ON010781** |
| *T. gamsii* | S488 | JN715613 | KJ665270 |
| *T. guizhouense* | S278 | KF134799 | KF134791 |
| *T. guizhouense* | **LZ056** | **ON597903** | **ON597876** |
| *T. hamatum* | CBS132565 | KJ665514 | KJ665275 |
| *T. hamatum* | S397 | JN715614 | JN715601 |
| *T. hamatum* | **LZ007** | **OM967096** | **OL957044** |
| *T. hamatum* | **LZ034** | **ON597891** | **ON597864** |
| *T. hamatum* | **LZ035** | **ON597892** | **ON597865** |
| *T. hamatum* | **LZ009** | **OM967076** | **ON010769** |
| *T. hamatum* | **LZ028** | **OM967077** | **ON010770** |
| *T. hamatum* | **LZ039** | **ON597893** | **ON597866** |
| *T. harzianum* | TRS55 | KP008803 | KP009121 |
| *T. harzianum* | TRS94 | KP008802 | KP009120 |
| *T. harzianum* | **LZ010** | **OM967080** | **ON010773** |
| *T. harzianum* | **LZ013** | **OM967081** | **ON010774** |
| *T. harzianum* | **LZ014** | **OL957045** | **OM967097** |
| *T. harzianum* | **LZ015** | **OM967082** | **ON010775** |
| *T. harzianum* | **LZ016** | **OM967083** | **ON010776** |
| *T. harzianum* | **LZ017** | **OM967084** | **ON010777** |
| *T. harzianum* | **LZ018** | **ON597894** | **ON597867** |
| *T. harzianum* | **LZ019** | **ON597895** | **ON597868** |
| *T. harzianum* | **LZ020** | **ON597896** | **ON597869** |
| *T. harzianum* | **LZ026** | **ON597897** | **ON597870** |
| *T. harzianum* | **LZ027** | **ON597898** | **ON597871** |
| *T. harzianum* | **LZ029** | **ON597899** | **ON597872** |
| *T. harzianum* | **LZ032** | **ON597900** | **ON597873** |
| *T. harzianum* | **LZ036** | **ON597901** | **ON597874** |
| *T. harzianum* | **LZ037** | **ON597902** | **ON597875** |
| *T. koningii* | S227 | KC285596 | JN715609 |
| *T. koningiopsis* | HZA6 | MK850828 | MH647796 |
| *T. koningiopsis* | S359 | KJ665546 | KJ665285 |
| *T. koningiopsis* | **LZ033** | **OL957048** | **OM967100** |
| *T. koningiopsis* | **LZ038** | **OM967089** | **ON010782** |
| *T. pleuroticola* | CBS 124383 | HM142381 | HM142371 |
| *T. pleuroticola* | TRS70 | KP008951 | KP009172 |
| *T. pleuroticola* | **LZ004** | **OM967103** | **OM967102** |
| *T. spirale* | TRS111 | KP008963 | KP009182 |
| *T. spirale* | **LZ022** | **OL957046** | **OM967098** |
| *T. spirale* | **LZ023** | **OM967085** | **ON010778** |
| *T. viride* | CBS119325 | DQ672615 | EU711362 |
| *T. viride* | TRS575 | KP008931 | KP009081 |
| *T. virens* | HZA14 | MK850836 | MH647804 |
| *T. virens* | **LZ001** | **OM967090** | **ON010783** |
| *T. virens* | **LZ012** | **OM967095** | **ON010788** |
| *T. virens* | **LZ045** | **ON597908** | **ON597881** |
| *T. virens* | **LZ052** | **ON597914** | **ON597887** |
| *T. virens* | **LZ046** | **ON597909** | **ON597882** |
| *T. virens* | **LZ055** | **ON597917** | **ON597890** |
| *T. virens* | **LZ002** | **OM967091** | **ON010784** |
| *T. virens* | **LZ003** | **OM967092** | **ON010785** |
| *T. virens* | **LZ008** | **OM967093** | **ON010786** |
| *T. virens* | **LZ011** | **OM967094** | **ON010787** |
| *T. virens* | **LZ030** | **ON597904** | **ON597877** |
| *T. virens* | **LZ031** | **ON597905** | **ON597878** |
| *T. virens* | **LZ044** | **ON597907** | **ON597880** |
| *T. virens* | **LZ047** | **ON597910** | **ON597883** |
| *T. virens* | **LZ048** | **ON597911** | **ON597884** |
| *T. virens* | **LZ049** | **ON597912** | **ON597885** |
| *T. virens* | **LZ050** | **ON597913** | **ON597886** |
| *T. virens* | **LZ051** | **OL957049** | **OM967101** |
| *T. virens* | **LZ052** | **ON597914** | **ON597887** |
| *T. virens* | **LZ053** | **ON597915** | **ON597888** |
| *T. virens* | **LZ054** | **ON597916** | **ON597889** |
| *T. virens* | **LZ055** | **ON597917** | **ON597890** |
| *T. viridescens* | S1 | KC285634 | KC285757 |
| *T. viridescens* | S452 | KC285646 | KC285758 |
